# Multi-Stress Monitoring System with Fiber-Optic Mandrels and Fiber Bragg Grating Sensors in a Sagnac Loop

**DOI:** 10.3390/s150818579

**Published:** 2015-07-29

**Authors:** Hyunjin Kim, Umesh Sampath, Minho Song

**Affiliations:** Division of Electronics and Information Engineering, Chonbuk National University, Jeonju 561-756, Korea; E-Mails: dldpavl@jbnu.ac.kr (H.K.); sjumesh@jbnu.ac.kr (U.S.)

**Keywords:** fiber Bragg grating, Sagnac interferometer, attenuator

## Abstract

Fiber Bragg grating sensors are placed in a fiber-optic Sagnac loop to combine the grating temperature sensors and the fiber-optic mandrel acoustic emission sensors in single optical circuit. A wavelength-scanning fiber-optic laser is used as a common light source for both sensors. A fiber-optic attenuator is placed at a specific position in the Sagnac loop in order to separate buried Bragg wavelengths from the Sagnac interferometer output. The Bragg wavelength shifts are measured with scanning band-pass filter demodulation and the mandrel output is analyzed by applying a fast Fourier transform to the interference signal. This hybrid-scheme could greatly reduce the size and the complexity of optical circuitry and signal processing unit, making it suitable for low cost multi-stress monitoring of large scale power systems.

## 1. Introduction

The power transformer is one of the most important pieces of equipment in electric power networks. Failures or mal-operations of power transformers cause all equipment that are connected to them to stop, which is one of the major concerns of the industrial and commercial electricity consumers as well as electric utilities [1,2,3]. In addition, failures of large power transformers are often accompanied by fire and/or spillage of environmentally hazardous fluid, which could harm people, other equipment, and local environment. Large power transformers are very expensive too. Considering all these issues, there is a clear motivation for utilities to maintain on-line monitoring of their power transformers.

There are different types of stresses which cause the failures of power transformers, such as electrical, electromagnetic, dielectric, thermal, and chemical stresses. In order to condition monitor the relevant parameters of these stresses and prevent accidents, a number of techniques have been studied and developed [2]. Of the techniques, due to the dielectric nature in high voltage environments, the fiber-optic sensors have attracted good interest widely. Fiber Bragg grating sensors were applied to find hot-spots within a transformer [4], and different types of fiber-optic interferometers have been used to measure acoustic signals [5]. Although the fiber-optic sensors have demonstrated good feasibility, the prototypes rarely have been commercialized and applied in the field. The main reasons could be attributed to the relatively high cost of the fiber-optic sensor system as well as the lack of skilled personnel who can handle the fiber-optics.

In this paper, we propose a hybrid fiber-optic sensor system that combines fiber grating sensors and fiber-optic mandrel sensors to measure temperature and acoustic signals simultaneously at different locations within a power transformer. The sensor system is able to greatly reduce the cost, the size, and the complexity because of the use of single fiber-optic circuit and signal processing system.

## 2. Hybrid Sensor System

### 2.1. Fiber-Optic Sensors

In this study, two kinds of fiber-optic sensors, the fiber Bragg grating (FBG) temperature sensors and the mandrel-based acoustic sensors, are employed in single optical circuit which uses a fiber-optic Sagnac interferometer. The different measurands change different parameters of the light passing through the Sagnac interferometer: the temperature changes the reflected Bragg wavelength and the acoustic wave changes the phase of the light passing through the mandrel sensor.

The Bragg wavelength shift can be measured by various FBG demodulation techniques. One of the simplest and the most widely-known techniques is the scanning band-pass filter demodulation which uses a broadband light source and a scanning Fabry-Perot filter [6]. In our previous study, we constructed an FBG sensor system with the demodulation technique to monitor the internal and the external temperature distributions of power transformers [7].

The other stress parameter to be measured with the hybrid sensor system is acoustic field. Acoustic emissions (AEs) in power transformers are usually generated by mechanical sources and electrical stresses, such as partial discharge and arcing. The levels of partial discharges (PDs) are indicators of the insulation condition, because they result in localized electrical breakdown that should not be present in significant values. Thus, the prognosis and diagnosis of insulation condition by detecting the AE has utmost importance [8].

Usually, the power transformers are filled with insulation oil where the hydrostatic pressure overwhelms the acoustic waves by many orders of magnitude. One way of measuring dynamic pressure is to sense the change in diameter of a compliant mandrel exposed to the dynamic pressure. A cylindrical body, possibly hollow, is often used as a compliant. The changing pressure ultimately produces a changing circumferential stress. At the same time, when an increase in pressure squeezes racially on the mandrel, the mandrel lengthens, that is, the mandrel experiences both axial and circumferential strains. Because of this sensitive reaction to dynamic pressure, this mandrel can be used as an AE sensor. These sensors are sometimes constructed by winding an optical fiber, under tension, around a deformable mandrel. Such a construction allows high sensitivity to pressure in a relatively small volume. The inherent dielectric nature and the high sensitivity of fiber-optic sensors enable them to measure AEs inside of the power transformer and find out the locations of the electrical stresses.

### 2.2. Fiber-Optic Sagnac Interferometer

The acoustic wave makes certain displacements in mandrel structure, modulating the optical path length of the fiber wound on mandrel’s surface. The changes in optical path length are transformed to the phase shifts of optical fields [8]. By analyzing the optical phase shifts, the acoustic waves can be reconstructed. The Sagnac interferometer is usually used to demodulate these optical phase shifts. The demodulation is based on the Sagnac effect that works as a basic working principle of fiber-optic gyro sensors [9,10].

**Figure 1:**
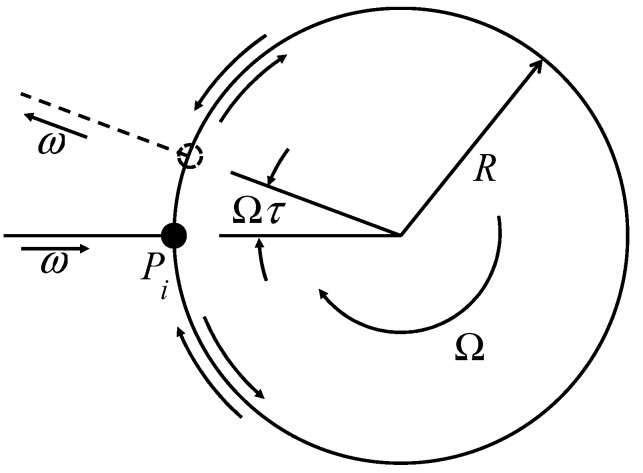
Sagnac effect in rotating circular loop.

In the Sagnac loop shown in Figure 1, the two waves propagating in opposite directions pass the same lengths until they exit the loop. Upon exiting the loop, the two waves combine, generating constructing interference. However, when the loop is rotating at Ω  (rad/s) of angular velocity, the clockwise and counterclockwise optical path lengths will be (2π+Ωτ)R and (2π−Ωτ)R, respectively, where the propagation time τ=2πR/c, c is the speed of light in vacuum, and R is the radius of Sagnac loop. The phase shift caused by the rotation can be described by following equation.

(1) $$\text{Δϕ}=\text{ωΔτ}=\text{ω}\frac{2\text{Ωτ}R}{c}=\frac{4\text{π}R^{2}\text{Ω}}{c^{2}}$$

This phase shift is transformed to sinusoidal intensity change in interference signal of Sagnac interferometer. By applying subsequent phase unwrapping techniques, the rotation of the Sagnac loop is calculated.

The fiber-optic mandrel sensor is based on almost the same principle. The dynamic pressure from the acoustic waves squeezes the mandrel sensor, and generates optical phase shift which is demodulated by the same technique with gyro sensors.

### 2.3. Hybrid Structure

**Figure 2:**
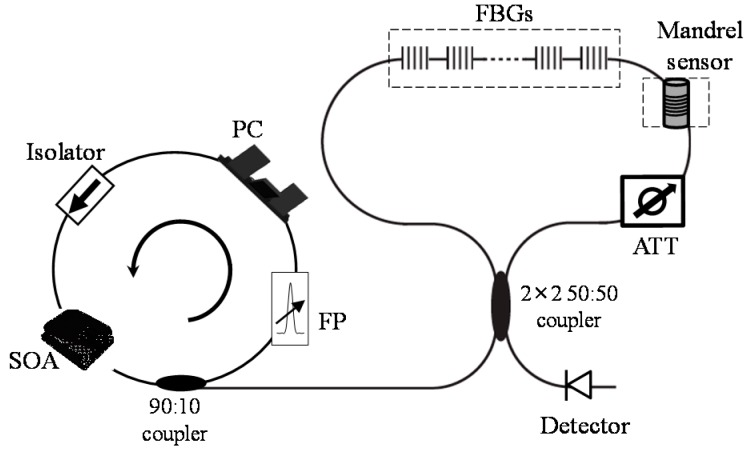
Schematic of the hybrid sensor system (SOA: semi-conductor optical amplifier, FP: Fabry-Perot wavelength filter, PC: polarization controller, ATT: attenuator).

Figure 2 is the schematic diagram of the suggested hybrid sensor system. FBG sensors and a mandrel sensor are placed in the same fiber-optic Sagnac loop. A broadband light source is considered to be the most suitable to address FBG sensors because of its simple and stable characteristics. The mandrel sensors, however, need to use a narrowband laser source for high intensity of interference signal. In the hybrid sensor system, in order to comply with the needs of both sensors’ we used a wavelength-scanning fiber-optic laser which was constructed with a Fabry-Perot wavelength filter, an optical semiconductor amplifier, an isolator, and a polarization controller.

**Figure 3:**
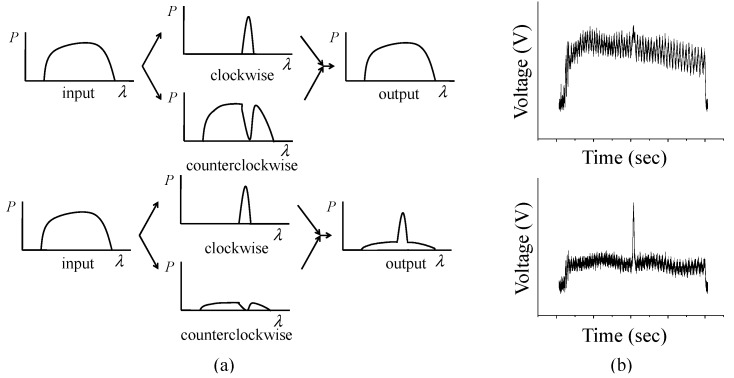
Sagnac interferometer outputs ((**a**) conceptual diagram without/with an attenuator (**b**) experimental photo-detector outputs without/with an attenuator).

When FBG sensors are placed in a Sagnac loop, it is difficult to demodulate the signals with scanning band-pass filter demodulation technique because they are buried in the interference signal as in Figure 3. In order to separate and demodulate the signals, we placed a fiber-optic attenuator at a specific location in the loop. With the attenuator in asymmetric position in the Sagnac loop, the intensity of transmitted spectrum attenuates dramatically and FBG signals stand out, which makes it possible to locate the central peaks of FBG sensors.

## 3. Experimental Results

In order to show the feasibility of suggested hybrid sensor system, we measured temperature and AE signal simultaneously by using FBG sensors and a mandrel sensor, respectively. The hybrid fiber-optic Sagnac loop was placed in an oil bath. A cylindrical piezoelectric transducer (PZT) was driven by a 200 kHz sine wave to simulate AE generated by partial discharge of which frequency range is known to be in 10~500 kHz [8].

**Figure 4:**
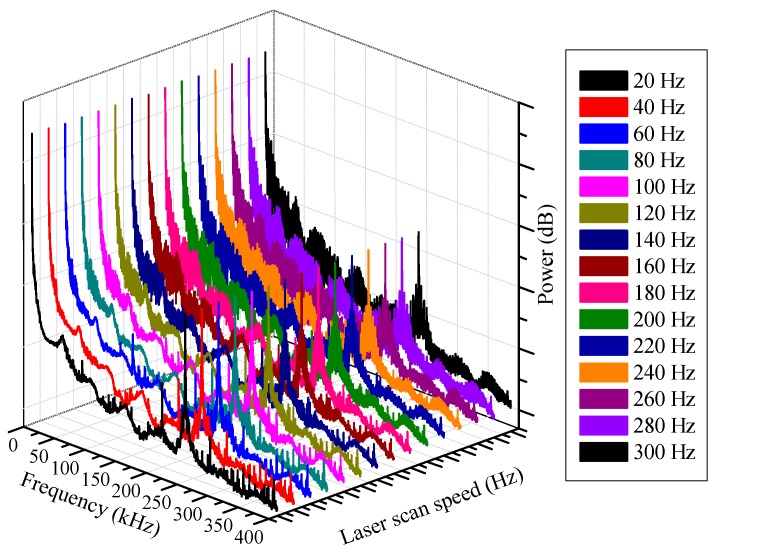
Output characteristics according to wavelength-scanning rate.

**Figure 5:**
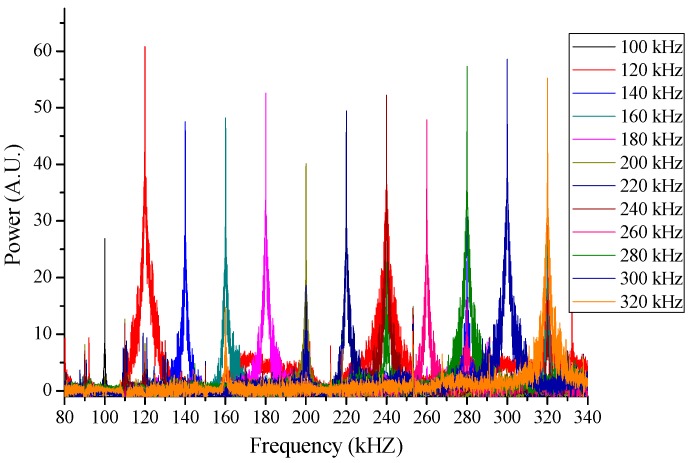
Measurement of various frequency components.

Figure 4 shows FFT spectra of interference signals which were measured at different wavelength-scanning rate of the laser source. As the wavelength-scanning rate increases, the output signal noise increased too. The signal-to-noise ratio of the FFT spectra dropped up to 6 dB while the scanning rate changed from 20 Hz to 300 Hz. Because the temperature measurement with this system does not require high bandwidth, we set the wavelength scanning rate at 20 Hz to minimize noise level. For further signal-to-noise improvement, FFT outputs were subtracted by a spectrum which had been obtained without PZT modulation. The enhanced FFT outputs of 100~320 kHz acoustic frequencies are shown in Figure 5 in 20 kHz step. All the frequency components in the range were detected successfully with minimum signal-to-noise ratio of 45 dB except at 100 kHz where background noise generated from the wavelength scanning and environmental effect is particularly larger than those at other frequencies.

**Figure 6:**
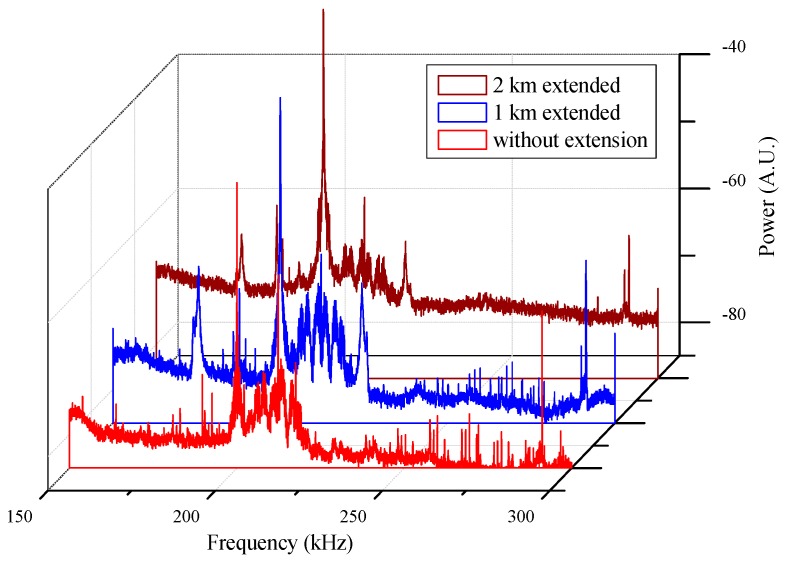
Acoustic emissions (AEs) measurements with different lengths of Sagnac loop.

Figure 6 shows AE analyses when different lengths of dummy fibers were inserted in the Sagnac loop. When the hybrid sensor system is used for condition monitoring of large scale power systems, the length of Sagnac loop could extend up to several kilometers. In order to prove the feasibility of measurement far away from the signal processing part, 1 and 2 km of optical fiber spools were connected to the loop. From the comparison of the experimental results, the background noise increased a little bit with addition of dummy fiber. However, the 200 kHz frequency component was successfully detected regardless of the length of Sagnac loop. This property could be used to increase the sensitivity of mandrel sensor by additional winding.

**Figure 7:**
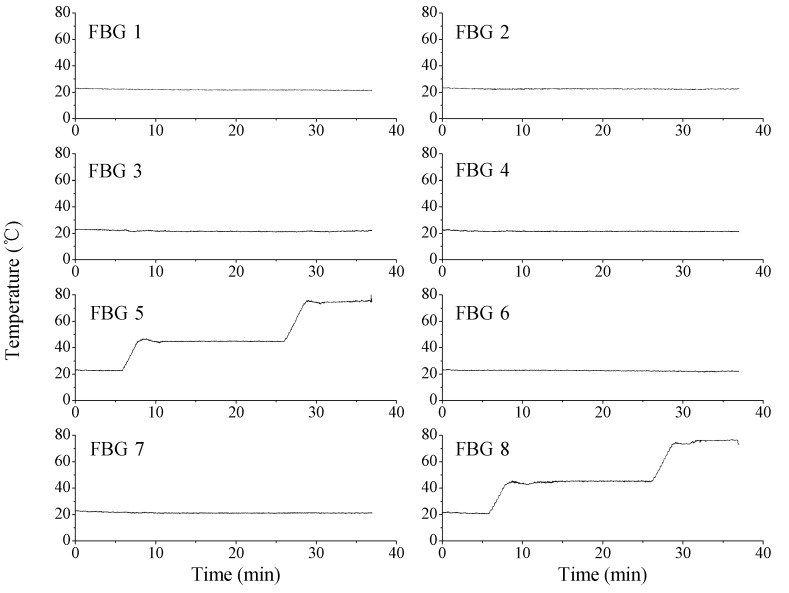
Temperature measurement with fiber Bragg gratings (FBGs).

During the AE measurement, the temperature distribution was also measured simultaneously. 8 FBGs were placed in the oil bath, and two of them were exposed to temperature change up to 80 °C. At room temperature, Bragg wavelengths were 1539.8, 1541.8, 1543.8, 1545.6, 1547.8, 1549.7, 1552.7, and 1554.7 nm. The Bragg wavelength shift against temperature change in the wavelength range was known to be about 0.01 nm/°C [11]. Figure 7 shows the measured temperature profiles of the FBG sensor array. Any crosstalk from the interference signal cannot be found in the FBG sensor outputs. With the 1 pm wavelength readout resolution we used [7], the temperature measurement resolution was about 0.1 °C, which was accurate enough for our applications in electric power systems.

## 4. Conclusions

We proposed a novel multi-stress fiber-optic sensor system for possible usage in condition monitoring of large scale electric power systems. The low signal-to-noise ratios of the FBG sensor signals were enhanced by using a wavelength-scanning laser and by placing a fiber-optic attenuator in the Sagnac loop. The temperature and the AE signals were successfully measured at the same time at multiple locations with the fiber-optic wavelength-scanning laser. The mandrel sensor could detect AE signal of 200 kHz even when the different lengths of optical fiber were added to Sagnac loop. This hybrid sensor system would be attractive as a quasi-distributive condition monitoring sensor system with low-cost and simple structure.
